# Effect of Processed Oil on Asphalt Binder Properties

**DOI:** 10.3390/ma15113739

**Published:** 2022-05-24

**Authors:** Navid Hemmati, Jihyeon Yun, Hyunhwan Kim, Moon-Sup Lee, Soon-Jae Lee

**Affiliations:** 1 Department of Engineering Technology, Texas State University, San Marcos, TX 78666, USA; n_h186@txstate.edu (N.H.); yiy1@txstate.edu (J.Y.); k_h82@txstate.edu (H.K.); sl31@txstate.edu (S.-J.L.); 2 Korea Institute of Civil Engineering and Building Technology, Goyang 10223, Korea

**Keywords:** processed oil, viscosity, rutting, fatigue, thermal cracking

## Abstract

This study investigates the effectiveness of processed oil in the modification of PG 64-22 and PG 76-22 by assessing their physical and rheological properties, and multiple comparison was conducted between the two binders. The base binders PG 64-22 and PG 76-22 were blended with processed oil at four different percentages of contents (3%, 6%, 9% and 12% by the weight of the binder) and compared with the control binder in each test. The base and modified binders were artificially short-term and long-term aged using a rolling thin film oven (RTFO) and pressure aging vessel (PAV) procedures. Superpave binder tests were performed on the modified binders by applying a rotational viscometer (RV), dynamic shear rheometer (DSR), and bending beam rheometer (BBR). The comparisons and results presented in this study indicate that (1) the processed oil has a significant effect on the binders’ viscosity, which changes with respect to the increment of processed oil content. The viscosity of both modified binders decreased with the addition of 3, 6, 9 and 12% processed oil; (2) the performed DSR test showed that the addition of processed oil had a negative effect on the rutting resistance for both binders, since in PG 64-22, G*/Sin *δ* values decreased by 55, 65, 75 and 83% with the addition of 3, 6, 9 and 12% processed oil, respectively, while a decrement of G*/Sin *δ* of 24, 45, 58 and 65% with the addition of 3, 6, 9 and 12% processed oil was observed in PG 76-22; meanwhile, the fatigue cracking performance was improved and was found to be effective, while G* Sin *δ* in PG76-22 decreased by 9, 30, 36, and 52% and in PG 64-22 by 27, 44, 53, and 67% with the addition of 3, 6, 9 and 12% processed oil; (3) the results from the BBR test indicate significant improvement in the thermal cracking properties of the binders. The addition of 3, 6, 9 and 12% processed oil resulted in a decrease in the stiffness of both the PG 64-22 and PG 76-22 binders, with a positive effect consequently being observed on the m-values of the binders.

## 1. Introduction

Asphalt binders are organic-based chemical mixtures, and are the most common materials used for paving roads and streets. Due to recent increases in traffic loads in cities, asphalt pavements are becoming increasingly susceptible to high- and low-temperature defects [1,2]. With the increased dynamic loads, in addition to the decrement of asphalt binder quality, the modification of asphalt binders is becoming increasingly critical. High-viscosity asphalt binders show good resistance against dynamic loads in terms of their viscoelasticity performance. However, this recoverable behavior is efficient within a specific range of temperatures. Additionally, asphalt binder viscosity decreases at higher temperatures, resulting in higher rutting, which may cause severe traffic accidents [3]. Asphalt binders exhibit linear and nonlinear viscoelastic behavior, depending on the stress and strain levels as a result of temperature [4].

On the other hand, thermal cracking occurs due to a loss of viscoelasticity in the asphalt binder’s molecular structure at low temperatures and under tensile stress and traffic load, especially in older pavements [5]. In accordance with the glass transition of asphalt binders at low temperatures, time-dependent hardening occurs due to changes in the asphalt’s molecular structure [6]. The viscosity of old asphalt binders increases due to the transition of aromatics to asphaltene components, resulting in higher stiffness or lower viscoelasticity and higher viscosity. In order to overcome these defects, modification of asphalt binders can be considered. However, due to the different performances of asphalt binders in the laboratory compared to on construction sites, the selection of the modifiers must made carefully. The modification of asphalt binders in most cases increases their viscosity; hence, the workability and handling of asphalt binders decreases in situ. Common modifiers that are widely employed in industry include Styrene–Butadiene–Styrene (SBS), Styrene–Isoprene–Styrene (SIS), Styrene–Butadiene–Rubber (SBR), Crumbed Rubber Modifier (CRM), and Polyethylene (PE) [7,8]. The mentioned additives increase the viscosity of asphalt binders; hence, the handling and pumping of the asphalt becomes more difficult. In addition, the increase in viscosity results in increased stiffness in asphalt binders, which can cause thermal cracking in asphalt pavement.

To overcome the effect of the high viscosity of modified asphalt binders, co-modifiers can play an important role. Oil compounds are highly effective materials that can alter the characteristics of asphalt binders [9]. Furthermore, studies have shown that the addition of different types of oil reduces the dynamic viscosity due to the high penetration rate [10,11]. On the other hand, applying oil in aged asphalt binder reduces the stiffness significantly due to the recovery of elasticity of the asphalt binder by rejuvenation [12,13]. Therefore, processed oil is considered one of the oil compounds that is effective for modifying the viscosity of asphalt binders during construction operations, and can additionally increase stiffness at low temperatures to avoid thermal cracking defects. However, limited studies have been performed to investigate the effects of different oil compounds, such as processed oil, on PG 64-22 and PG 76-22 asphalt binders in different temperature ranges.

**Figure 1:**
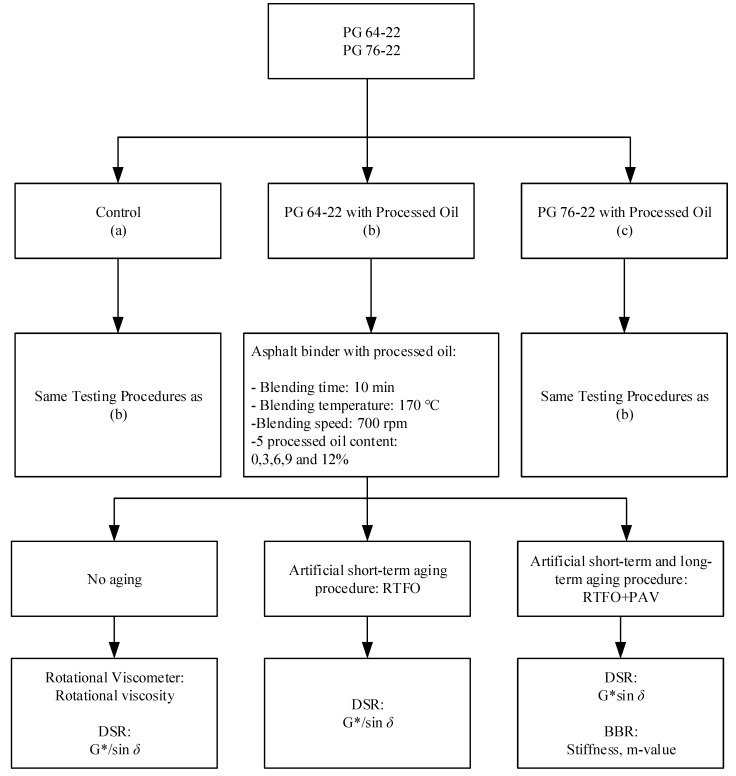
Flow chart of experimental design procedures.

The purpose of this study is to investigate the applicability of processed oil in asphalt binders and to determine its impact on viscosity and stiffness. In addition, this study is expanded to survey the ways in which it might be possible to use processed oil in conjunction with other polymers. In particular, the rheological properties of asphalt binders using processed oil are investigated after artificial short-term aging treatment in a rolling thin film oven (RTFO) and long-term aging treatment in a pressure aging vessel (PAV). First, the high-temperature viscosity of the binders was evaluated in the original state via a rotational viscometer (RV) test using four different temperatures ranging from 135 °C to 180 °C in 15 °C intervals. Next, the samples’ rutting performance was assessed using DSR in the original state, in the short-term aged state after RTFO, and in the long-term aged state after RTFO+PAV. Finally, the low-temperature cracking temperature was tested by BBR after RTFO+PAV. A flow chart of the experimental design used in this research is presented in Figure 1.

## 2. Experimental Design

### 2.1. Materials

Asphalt binders with performance grade (PG) 64-22 and 76-22 were used for this research. Both base binders are commonly applied in the pavement industry. The binders’ properties are shown in Table 1. Processed oil, which is a byproduct of distillation towers in petroleum refineries, can be used as an additive to the binders to decrease their viscosity while also increasing their viscoelasticity. From an industrial point of view, the application of processed oil can improve the binder’s workability and low-temperature performance in situ. Processed oil is a dark viscous liquid that is applicable for different purposes, such as lubricating industrial mechanical equipment. The characteristics of the processed oil considered in this study are presented in Table 2.

### 2.2. Production and Sample Preparation of Processed Oil Asphalt Binders

The binders were blended using a wet process, in which the processed oil was added directly to the base binder at the mixing temperature of 170 ± 5 °C. The blending duration was 10 min at a mixing speed of 700 rpm in the laboratory [14]. The mixing temperature was controlled manually and constantly to avoid approaching the boiling point of the asphalt binder and processed oil, which could affect the quality of samples. Both binders were modified with four different percentages of processed oil content (3, 6, 9, and 12% by weight of the binder). The same batch of processed oil was used in order to maintain consistency in the experiments.

**Figure 2:**
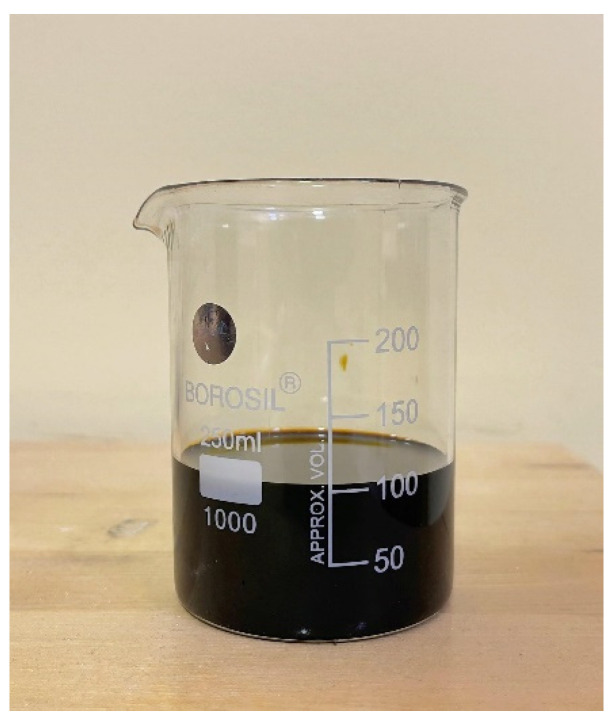
Processed oil.

Artificial aging processes were performed for the control and processed-oil-modified binders. These consisted of 85 min at 163 °C to achieve short-term aging, followed by PAV for 20 h at 100 °C at a pressure of 2 bar to achieve long-term aging. After completing the aging processes, the tests mentioned in Figure 1 were conducted to investigate the properties of the processed-oil-modified binders. Figure 2 illustrates the appearance of processed oil.

#### 2.2.1. Basic Characteristics Tests

A rotational viscosity test was performed at temperatures ranging from 135 °C to 180 °C in 15 °C intervals while applying a constant velocity of 20 rpm to investigate the binders’ workability, which is one of the most important factors in special asphalt mixtures. The binders were scaled to 10.5 g and the test was performed using 27 cylindrical spindles. A duration of 20 min was considered for each sample to collect rotational viscosity data.

To investigate the viscoelasticity of asphalt binders, the G*/sin *δ* was obtained from the complex shear modulus (G*) and the Sine of the phase angle (*δ*) at 82 °C. By employing the factors of G* and (*δ*) at a lower temperature of 25 °C, the fatigue cracking resistance of the asphalt binders was evaluated.

The stiffness of the binders was evaluated at −12 °C and −24 °C using Bending Beam Rheometer (BBR; Canon, USA) equipment according to the AASHTO T 313. The specimens were shaped by pouring them into beam molds (125 × 6.35 × 12.7 mm^3^) and placing them in a bath to measure the creep stiffness of each of them with a load mass of 100 g over a duration of 60 s.

#### 2.2.2. Statistical Analysis Method

The Statistical Package for the Social Sciences program (SPSS) was used to carry out statistical analysis of variance (ANOVA) and Fisher’s Least Significant Difference (LSD) comparison with an α = 0.05. The major variables included the processed oil content (0, 3, 6, 9 and 12%) and the asphalt binder type (PG 64-22 and PG 76-22). ANOVA was performed first to investigate whether significant differences existed among samples’ means. In this study, the significance level was 95 (α = 0.05), suggesting that each finding had a 95% chance of being true. Using ANOVA, the LSD was calculated after determining that the means of the samples were different. LSD is calculated as the difference between two sample means necessary to determine the difference between two populations. Once the LSD had been calculated, all pairs of samples mean were compared. In the case of difference between two sample means greater than or equal to the LSD, the population means were determined as being statistically different.

## 3. Result and Discussion

### 3.1. Rotational Viscosity

**Figure 3:**
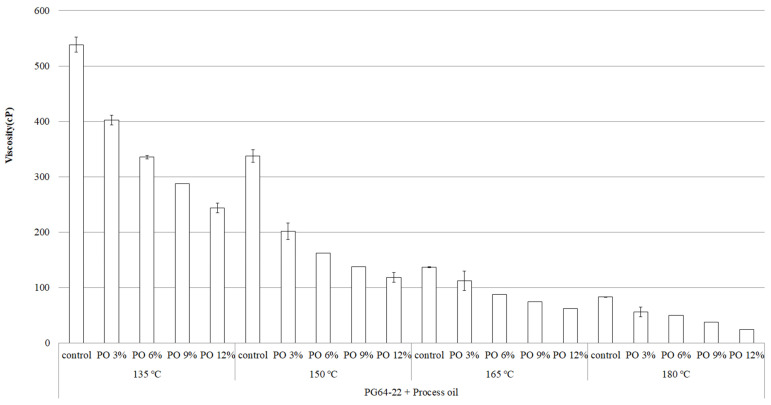
Viscosity of PG 64-22 binder as a function of processed oil content and temperature increment.

**Figure 4:**
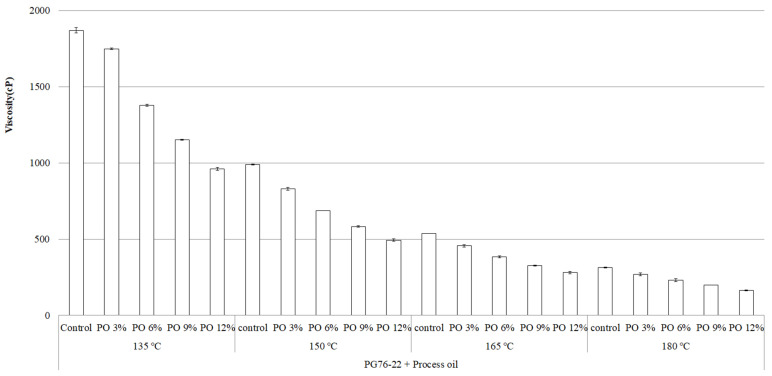
Viscosity of PG 76-22 binder as a function of processed oil content and temperature increment.

The determination of mixing and compaction temperatures for asphalt binders is vital to achieving the best quality in terms of the handling conditions, workability, coating of aggregates, and compaction to a new asphalt surface [15]. Figure 3 and Figure 4 illustrate the standard RV test results of PG 64-22 and PG 76-22 (original and modified) at 135 °C, 150 °C, 165 °C, and 180 °C. Figure 3 shows that with the addition of processed oil, the viscosity of PG 64-22 decreases significantly with increasing temperature. At 135 °C, the viscosity values of PG 64-22 containing 0, 3, 6, 9, and 12% processed oil were observed to be 538, 400, 335, 287, and 243 cP, respectively. As shown in Figure 4, with the same oil concentrations of PG 76-22 at 135 °C, the viscosity results obtained were 1870, 1750, 1380, 1150, and 960 cp, respectively. With the increment of processed oil content to 3, 6, 9, and 12%, the viscosity of PG 64-22 modified binders decreased by 25, 38, 47, and 55%, whereas in PG 76-22 modified binders, was found to decrease by 7, 27, 39, and 49%, respectively. Subsequently, at temperatures of 150 °C and 165 °C, a similar decreasing trend was observed for viscosity with the addition of processed oil. At 180 °C, decreases in the viscosity value of 33, 41, 56, and 71% were observed for PG 64-22 at processed oil contents of 3, 6, 9, and 12%. On the other hand, for PG 76-22 at the same temperature and the same content of processed oil, the viscosity decreased by 15, 27, 37, and 48%, indicating a lower slope of decrement compared to PG 64-22. The observed results show that, due to the alternation of processed oil molecules in the asphaltene molecular chain, higher processed oil contents result in lower viscosity.

The obtained results show that the addition of processed oil decreases the viscosity of the asphalt binders due to the high penetration and restructuring of asphaltene molecules, which results in a significant rejuvenation of the asphalt binders. The effect of processed oil is more evident in PG 64-22 rather than in PG 76-22, indicating that low-viscosity binders are advantageous when aiming to reduce viscosity using processed oil. The reduction in viscosity makes it possible to improve the workability conditions, including pumping, coating, and compaction, especially in conjunction with some high-viscosity modified binders (e.g., styrene–butadiene–rubber (SBR), crumbed rubber modifier (CRM), styrene–butadiene–styrene (SBS)). Both Figure 3 and Figure 4 show a decreased viscosity that is in accordance with the Superpave specifications (Asphalt Institute 2003), which specify viscosity much lower than 3000 cp at temperatures below 135 °C, and it seems that the viscosity can be kept low even with the co-addition of other polymers.

The statistical significance of the change in viscosity as a function of processed oil content, temperature change, and binder type were examined, and the results are summarized in Table 3 and Table 4. It was observed at each temperature that the changes were significant for both PG 64-22 and PG 76-22 binders at the tested temperatures. This means that the impact of processed oil on viscosity is statistically significant at all of the tested temperatures, regardless of the type of binders.

### 3.2. Rutting Properties

**Figure 5:**
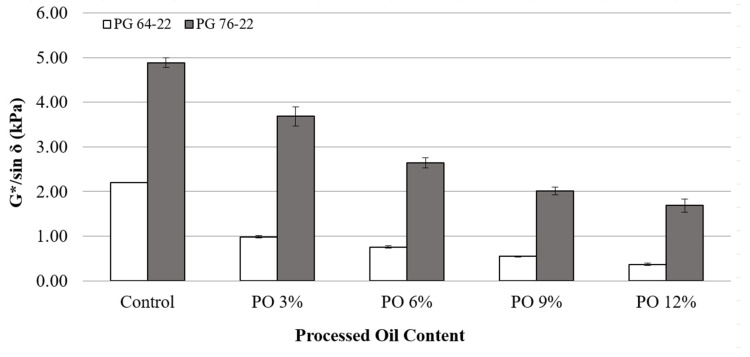
G*/sin *δ* of the original asphalt binders with processed oil at 64 °C.

In general, a higher G*/sin *δ* value obtained from the DSR test means that the binders are more effective against rutting at high pavement temperatures (Asphalt Institute 2003). The G*/sin *δ* values of binders were tested at the original state and the short-term aging (RTFO) state at 64 °C. Figure 5 depicts the results of the conducted tests of the asphalt binders in the original state. The addition of processed oil decreased the G*/sin *δ* values for both binder types. This means that the addition of processed oil alone negatively influences the rutting resistance, which was predictable due to the decrement of the viscosity. The G*/sin *δ* values of PG 64-22 decreased by 55, 65, 75 and 83% due to addition of 3, 6, 9 and 12% processed oil content. For PG 76-22 the values of G*/sin *δ* were observed decreased by 24, 45, 58 and 65% due to the addition of 3, 6, 9 and 12% processed oil. According to the Superpave specifications (Asphalt Institute 2003), G*/sin *δ* of all unaged binders must remain above 1.00 kPa to prevent rutting at each temperature. Even though base PG 64-22 showed a higher G*/sin *δ* value than 1.00 kPa, all binders containing the processed oil resulted in lower G*/sin *δ* below 1. Therefore, the PG 76-22 with processed oil might fail at higher testing temperatures though all of them passed it at 64 °C. Processed oil can adequately conjugate other modifiers like SBR, SBS, or CRM to secure acceptable quality in rutting resistance considering the site condition.

Table 5 illustrates the changes of G*/sin *δ* as a function of processed oil content for both PG 64-22 and PG 76-22. All G*/sin *δ* values of base binders (PG 64-22 and PG 76-22) showed statistically significant results depending on the processed oil contents. It means that the effect of processed oil on diminishing the rutting resistance is apparent.

**Figure 6:**
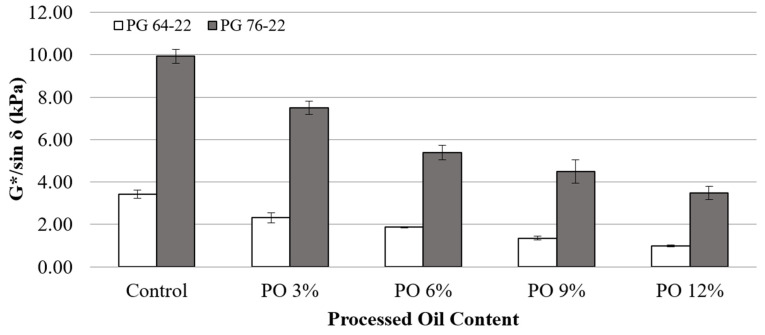
G*/sin *δ* of the RTFO asphalt binders with processed oil at 64 °C.

Figure 6 illustrates the values of G*/sin *δ* for both binder types of PG 64-22 and PG 76-22 in a short-term aging state (RTFO). A similar decrement trend was observed for this condition as before aging. The samples were tested at 64 °C and compared to unaged binders. The application of processed oil transformed the molecular structure of the asphalt binders and decreased the viscosity. The G*/sin *δ* values decreased by 32, 45, 60, and 71% as processed oil content increased with 3% interval from 3% to 12%. On the other hand, with the same values of processed oil for PG 76-22, the G*/sin *δ* values decreased by 24, 45, 54, and 65%, respectively. In both binders, under short-term aging occasions, the results showed a decrement of viscosity because of rejuvenating the asphalt binder via processed oil.

Table 6 shows the statistical analysis of changes due to the addition of processed oil in both binder types. In general, significant differences in G*/sin *δ* results of both base binders were observed depending on oil content, except for results between 3% and 6% and 9% and 12%, indicating the effect of processed oil on rutting performance is practical even in short-term aged binders.

### 3.3. Fatigue Cracking

**Figure 7:**
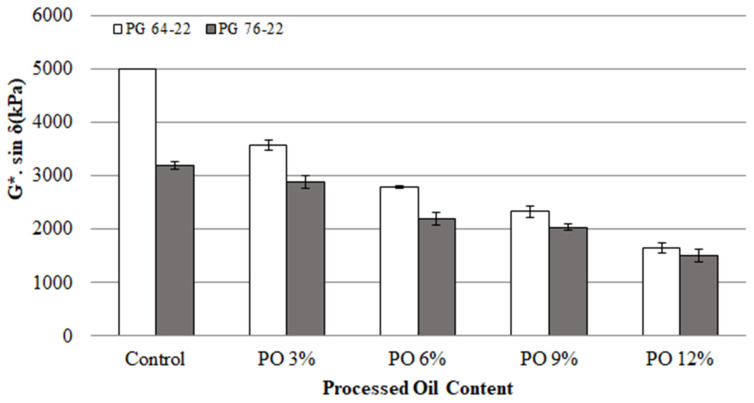
G*sin *δ* of the RTFO + PAV asphalt binders with processed oil at 25 °C.

The G*sin *δ* values of the binders (RTFO+PAV residual) obtained from the DSR test represent their fatigue cracking characteristics, where G* represents stiffness and *δ* is a viscosity or elasticity indicator. To determine G*sin *δ*, a rheology test was conducted at 25 °C for the modified samples of both binder types. Figure 7 illustrates the G*sin *δ* values of all binders. The G* sin *δ* values of the modified binders of PG 64-22 containing 0, 3, 6, 9, and 12% processed oil were observed to be 5000, 3570, 2790, 2333, and 1653 kPa, respectively, while for PG 76-22 with the same processed oil content, values of 3186, 2893, 2203, 2030, and 1503 kPa, respectively, were observed. As a result of the addition of processed oil to PG 64-22, the G*sin *δ* decreased by 27, 44, 53, and 67%, respectively. On the other hand, as a result of the addition of processed oil to PG 76-22, the G*sin *δ* values decreased by 9, 30, 36, and 52%. As is to be expected, the addition of processed oil decreases the viscosity, positively affecting fatigue cracking resistance, as observed in Figure 7.

Table 7 presents the statistical analysis of the changes in G* sin *δ* as a function of processed oil content. The addition of processed oil showed significant differences between different contents in both PG 64-22 and PG 76-22 binder types, meaning that the addition of processed oil is statistically effective for improving fatigue cracking resistance.

### 3.4. Low-Temperature Cracking Properties

**Figure 8:**
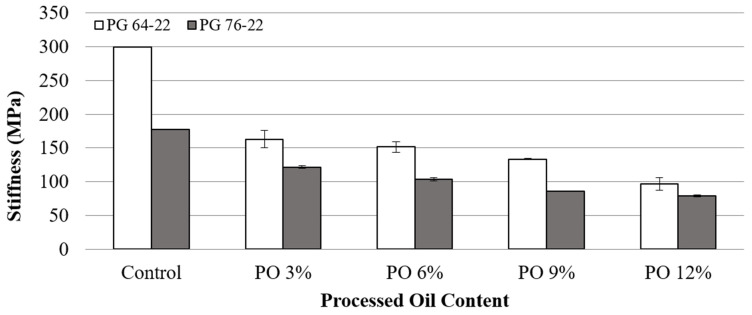
Stiffness of asphalt binder with processed oil at −12 °C.

**Figure 9:**
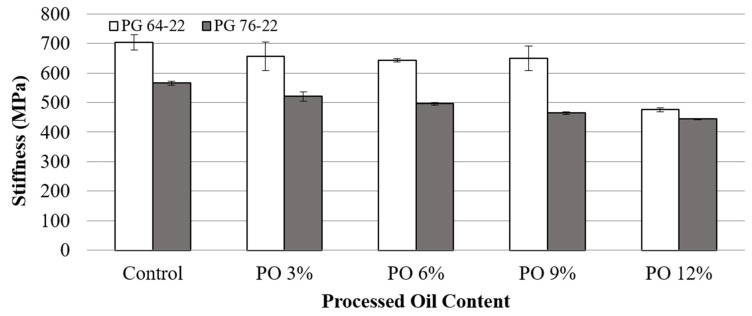
Stiffness of asphalt binder with processed oil at −24 °C.

The Superpave asphalt binder specifications indicate creep stiffness values up to a maximum of 300 MPa. The reduction in stiffness causes a decrease in the tensile stress of the asphalt binder, which means less possibility of low-temperature cracking. Therefore, BBR tests were conducted at −12 °C and −24 °C to characterize the low-temperature cracking properties of the modified binders. Figure 8 and Figure 9 show the stiffness values of the modified binders at temperatures of −12 °C and −24 °C. At −12 °C, the stiffness values for PG 64-22 with oil contents of 0, 3, 6, 9, and 12% were determined to be 300, 163, 152, 133, and 96 MPa. This shows that with each 3% addition of processed oil, the stiffness decreased by 46, 7, 12, and 28%, respectively. Additionally, PG 76-22 binder containing the same processed oil contents exhibited a decreasing trend of stiffness, with reductions in stiffness of 32, 14, 17, and 8% with each 3% addition of processed oil. However, the stiffness results measured at −24 °C showed low reduction percentages, which were less than 10%, except for the 12% oil content of PG 64-22. On the basis of these results, it can be concluded that the effect of oil on asphalt binders decreases at a lower temperatures due to nearing the freezing point of oil, even though the addition of oil is apparently effective at reducing the stiffness of binders. Additionally, PG 76-22 showed lower stiffness values than PG 64-22 for all oil contents. However, it is believed that the addition of oil can equal the cracking resistance of polymer-modified binders, as both base binders with the addition of 12% processed oil presented reasonably similar values.

**Figure 10:**
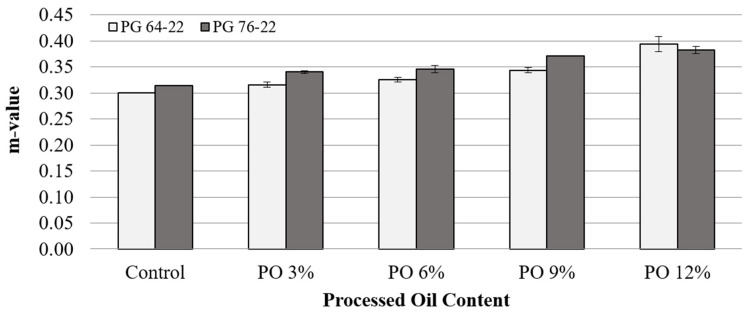
m-value of asphalt binder with processed oil at −12 °C.

**Figure 11:**
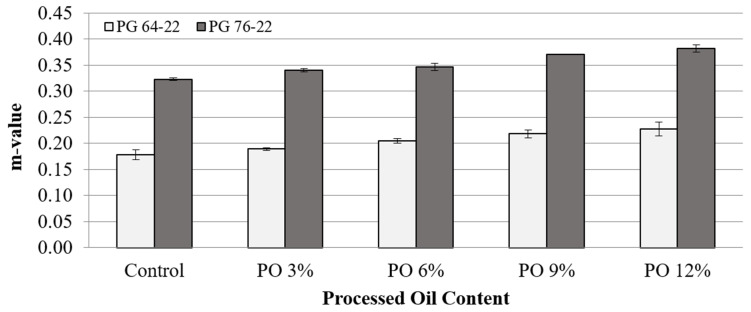
m-value of asphalt binder with processed oil at −24 °C.

Figure 10 and Figure 11 illustrate the m-values of the PG 64-22 and PG 76-22 asphalt binders at temperatures of −12 °C and −24 °C. A very slight increase in m-values was observed in both asphalt binders at −12°C, which was to be expected, considering the stiffness results mentioned before. This tendency was also observed in the results at −24 °C. On the basis of these results, it is thought that processed oil is effective for improving the thermal cracking property by increasing the m-value. As shown in Figure 10 and Figure 11, the addition of processed oil rejuvenates the asphalt and makes it flexible. Thus, the asphalt binders are less susceptible to thermal cracking with the addition of processed oil. In particular, the m-value of PG 76-22 at −24 °C was higher than 0.3, which is the minimum value required by Superpave, even though the stiffness result of PG 76-22 did not satisfy this standard, which cites a maximum value of 300 MPa. This is not believed to be due to the impact of the processed oil, but rather due to the characteristics of the original PG 76-22 binder used.

The statistical analysis of changes in stiffness as a function of processed oil content at temperatures of −12 °C and −24 °C is shown in Table 8 and Table 9, respectively. In general, asphalt modification resulted in a significant difference at a temperature of −12 °C. However, insignificant differences were observed for each oil content at a temperature of −24 °C. This indicates that processed oil is not statistically effective for improving cracking resistance at −24 °C compared to the results at −12 °C.

Table 10 and Table 11 illustrate the significance of changes in m-value due to the addition of processed oil for both asphalt binders at −12 °C and −24 °C. Compared to the stiffness results, which showed an insignificant effect of the addition of processed oil at −24 °C, these results generally indicate a significant difference in m-value depending on the oil content. As mentioned before, the impact of the addition of processed oil on increasing the m-value is verified based on statistical analysis.

## 4. Summary and Conclusions

To examine the effect of the addition of processed oil on asphalt binders, the original binder was artificially aged using short-term and long-term aging. The tests were conducted using rotational viscosity, a dynamic shear rheometer, and a bending beam rheometer in order to evaluate the properties of the binders. Based on the results, the following conclusions can be drawn about the effects of processed oil in this study.

1. The addition of processed oil reduced the viscosity at all testing temperatures, as expected. Two base binders containing processed oil appeared to have a lower viscosity than the control binders with no oil. Therefore, it is thought that the addition of processed oil improves workability, including pumping and compaction.
2. On the basis of the DSR test, it was observed that increasing the processed oil content made it possible to reduce G*/sin *δ* in both original and short-term aging conditions. Moreover, G*/sin *δ* gradually decreased with increasing oil content. Based on these results, the application of processed oil negatively affects the rutting resistance in both aging states.
3. The value of G*sin *δ* for the processed-oil-modified binders was observed to have a decreasing trend with increasing oil content. Moreover, this was an almost proportionally increasing trend with increasing oil content. Therefore, processed oil is found to be effective at improving fatigue cracking performance for both binder types.
4. The results of the BBR test showed that incorporating processed oil made it possible to steadily decrease the stiffness. Additionally, the addition of processed oil showed a positive effect on the m-value with increasing oil content. These results verified that processed oil plays a role in increasing thermal cracking resistance at low temperatures.
5. The application of processed oil generally impacted binder performances, including rutting and cracking. The addition of processed oil had an undesirable effect on rutting, while the cracking performances were considerably improved. Therefore, it is necessary to conduct deeper analysis to figure out the properties of processed oil when incorporating various polymer additives. Future studies would be desirable in order to investigate the effects of the addition of processed oil in conjunction with common modifiers (e.g., SBR, SBS, SIS, CRM). Additionally, it is necessary to conduct experimental tests on asphalt mixtures modified using processed oil.

## Figures and Tables

**Table 1:** Properties of asphalt binders (PG 64-22 and PG 76-22).

Aging States	Test Properties	Standard	PG 64-22	PG 76-22
No aging binder	Viscosity @ 135 °C (cP)	AASHTO PP6	538	1870
	G*/sin *δ* @ 64 °C (kPa)	AASHTO PP6	2.2	4.8
RTFO aged residual	G*/sin *δ* @ 64 °C (kPa)	AASHTO PP6	3.4	9.9
RTFO+PAV aged residual	G*sin *δ* @ 25 °C (kPa)	AASHTO PP6	5000	3186
	Stiffness @ −12 °C (MPa)	AASHTO PP6	300	177
	m-value @ −12°C	AASHTO PP6	0.300	0.314

**Table 2:** Properties of processed oil.

Test Item	Condition	Unit	Result	Standard
Specific gravity	@ 15 °C		1.0049	ASTM D4052
Flash Point	COC	°C	228	ASTM D 92
Kinematic Viscosity	@98.9 °C	mm^2^/S	22.59	ASTM D 445
	@100 °C	mm^2^/S	21.53	ASTM D 445
Pour point		°C	+15	ASTM D 97
Viscosity Gravity Constant			0.9555	ASTM D 2140
Hydrocarbon type	Ca (Aromatic)	%	41.9	ASTM D 2140
	Cn (Naphtenic)	%	26.2	ASTM D 2140
	Cp (paraffinic)	%	31.9	ASTM D 2140

**Table 3:** Statistical analysis results of the RV test for PG 64-22 as a function of processed oil content (α = 0.05).

Viscosity		135 °C					150 °C					165 °C					180 °C				
	PO%	0	3	6	9	12	0	3	6	9	12	0	3	6	9	12	0	3	6	9	12
135 °C	0	-	S	S	S	S	S	S	S	S	S	S	S	S	S	S	S	S	S	S	S
	3		-	S	S	S	S	S	S	S	S	S	S	S	S	S	S	S	S	S	S
	6			-	S	S	N	S	S	S	S	S	S	S	S	S	S	S	S	S	S
	9				-	S	S	S	S	S	S	S	S	S	S	S	S	S	S	S	S
	12					-	S	S	S	S	S	S	S	S	S	S	S	S	S	S	S
150 °C	0						-	S	S	S	S	S	S	S	S	S	S	S	S	S	S
	3							-	S	S	S	S	S	S	S	S	S	S	S	S	S
	6								-	S	S	S	S	S	S	S	S	S	S	S	S
	9									-	S	N	S	S	S	S	S	S	S	S	S
	12										-	S	N	S	S	S	S	S	S	S	S
165 °C	0											-	S	S	S	S	S	S	S	S	S
	3												-	S	S	S	S	S	S	S	S
	6													-	S	S	N	S	S	S	S
	9														-	S	N	S	S	S	S
	12															-	S	N	S	S	S
180 °C	0																-	S	S	S	S
	3																	-	N	S	S
	6																		-	S	S
	9																			-	S
	12																				-

S: significant; N: non-significant.

**Table 4:** Statistical analysis results of the RV test for PG 76-22 as a function of processed oil content (α = 0.05).

Viscosity		135 °C					150 °C					165 °C					180 °C				
	PO%	0	3	6	9	12	0	3	6	9	12	0	3	6	9	12	0	3	6	9	12
135 °C	0	-	S	S	S	S	S	S	S	S	S	S	S	S	S	S	S	S	S	S	S
	3		-	S	S	S	S	S	S	S	S	S	S	S	S	S	S	S	S	S	S
	6			-	S	S	S	S	S	S	S	S	S	S	S	S	S	S	S	S	S
	9				-	S	S	S	S	S	S	S	S	S	S	S	S	S	S	S	S
	12					-	S	S	S	S	S	S	S	S	S	S	S	S	S	S	S
150 °C	0						-	S	S	S	S	S	S	S	S	S	S	S	S	S	S
	3							-	S	S	S	S	S	S	S	S	S	S	S	S	S
	6								-	S	S	S	S	S	S	S	S	S	S	S	S
	9									-	S	S	S	S	S	S	S	S	S	S	S
	12										-	S	S	S	S	S	S	S	S	S	S
165 °C	0											-	S	S	S	S	S	S	S	S	S
	3												-	S	S	S	S	S	S	S	S
	6													-	S	S	S	S	S	S	S
	9														-	S	S	S	S	S	S
	12															-	S	S	S	S	S
180 °C	0																-	S	S	S	S
	3																	-	S	S	S
	6																		-	S	S
	9																			-	S
	12																				-

S: significant N: non-significant.

**Table 5:** Statistical analysis results of the G*/sin *δ* as a function of processed oil content (α = 0.05).

G*/Sin *δ*	PO %	0	3	6	9	12
PG 64-22	0	-	S	S	S	S
	3		-	S	S	S
	6			-	S	S
	9				-	S
	12					-
PG 76-22	0	-	S	S	S	S
	3		-	S	S	S
	6			-	S	S
	9				-	S
	12					-

S: significant N: non-significant.

**Table 6:** Statistical analysis results of the G*/sin *δ* of RTFO test as a function of processed oil content (α = 0.05).

G*/Sin *δ*	PO %	0	3	6	9	12
PG 64-22	0	-	S	S	S	S
	3		-	N	S	S
	6			-	S	S
	9				-	N
	12					-
PG 76-22	0	-	S	S	S	S
	3		-	S	S	S
	6			-	S	S
	9				-	S
	12					-

S: significant N: non-significant.

**Table 7:** Statistical analysis results of the G*sin *δ* as a function of processed oil content (α = 0.05).

G* Sin *δ*	PO %	0	3	6	9	12
PG 64-22	0	-	S	S	S	S
	3		-	S	S	S
	6			-	S	S
	9				-	S
	12					-
PG 76-22	0	-	S	S	S	S
	3		-	S	S	S
	6			-	S	S
	9				-	S
	12					-

S: significant N: non-significant.

**Table 8:** Statistical analysis results of the stiffness as a function of processed oil content at −12 °C (α = 0.05).

Stiffness	PO %	0	3	6	9	12
PG 64-22	0	-	S	S	S	S
	3		-	N	S	S
	6			-	S	S
	9				-	S
	12					-
PG 76-22	0	-	S	S	S	S
	3		-	N	S	S
	6			-	S	S
	9				-	N
	12					-

S: significant N: non-significant.

**Table 9:** Statistical analysis results of the stiffness as a function of processed oil content −24 °C (α = 0.05).

Stiffness	PO %	0	3	6	9	12
PG 64-22	0	-	N	S	S	S
	3		-	N	N	S
	6			-	N	S
	9				-	S
	12					-
PG 76-22	0	-	N	S	S	S
	3		-	N	S	S
	6			-	N	S
	9				-	N
	12					-

S: significant N: non-significant.

**Table 10:** Statistical analysis results of the m-value as a function of processed oil content −12 °C (α = 0.05).

m-Value	PO %	0	3	6	9	12
PG 64-22	0	-	S	S	S	S
	3		-	N	S	S
	6			-	S	S
	9				-	S
	12					-
PG 76-22	0	-	S	S	S	S
	3		-	N	S	S
	6			-	S	S
	9				-	N
	12					-

S: significant N: non-significant.

**Table 11:** Statistical analysis results of the m-value as a function of processed oil content −24 °C (α = 0.05).

m-Value	PO %	0	3	6	9	12
PG 64-22	0	-	N	S	S	S
	3		-	S	S	S
	6			-	S	S
	9				-	S
	12					-
PG 76-22	0	-	S	S	S	S
	3		-	N	S	S
	6			-	S	S
	9				-	N
	12					-

S: significant N: non-significant.

## Data Availability

The data used to support the findings of this study are included within the article.

## References

[1] Chopra T., Parida M., Kwatra N., Chopra P. (2018). Development of pavement distress deterioration prediction models for urban road network using genetic programming. Adv. Civ. Eng. Civ. Eng..

[2] He D., Yang W. (2018). Effect of thickness of gravel base and asphalt pavement on road deformation. Adv. Civ. Eng..

[3] Li N., Zhan H., Yu X., Tang W., Yu H., Dong F. (2021). Research on the high temperature performance of asphalt pavement based on field cores with different rutting development levels. Mater. Struct..

[4] Garba R. Permanent Deformation Properties of Asphalt Concrete Mixtures. https://ntnuopen.ntnu.no/ntnu-xmlui/handle/11250/231328.

[5] Tabatabaee H.A., Velasquez R., Bahia H.U. (2012). Predicting low temperature physical hardening in asphalt binders. Constr. Build. Mater..

[6] Zhou W.Y., Chen Z.G., Chen Z.N., Qin W.J., Yi J.Y. (2021). Generation and propagation of reflective cracking and thermal cracking of asphalt pavement in cold regions of China. Eleventh International Conference on the Bearing Capacity of Roads, Railways and Airfields.

[7] Halle M., Rukavina T., Domitrovic J. Influence of temperature on asphalt stiffness modulus. Proceedings of the 5th Eurasphalt Eurobitume Congress.

[8] Kim H.H., Mazumder M., Lee S.J., Lee M.S. (2017). Characterization of recycled crumb rubber modified binders containing wax warm additives. J. Traffic Transp. Eng..

[9] Kim H.H., Mazumder M., Torres A., Lee S.J., Lee M.S. (2017). Characterization of CRM binders with wax additives using an atomic force microscopy (AFM) and an optical microscopy. Adv. Civ. Eng. Mater..

[10] Borhan M.N., Suja F., Ismail A., Rahmat RA O.K. (2009). The effects of used cylinder oil on asphalt mixes. Eur. J. Sci. Res..

[11] Woszuk A., Wróbel M., Franus W. (2019). Influence of waste engine oil addition on the properties of zeolite-foamed asphalt. Materials.

[12] Sonibare K., Rucker G., Zhang L. (2021). Molecular dynamics simulation on vegetable oil modified model asphalt. Constr. Build. Mater..

[13] Shoukat T., Yoo P.J. (2018). Rheology of asphalt binder modified with 5W30 viscosity grade waste engine oil. Appl. Sci..

[14] Król J.B., Khan R., Collop A.C. (2018). The study of the effect of internal structure on permeability of porous asphalt. Road Mater. Pavement Des..

[15] Asphalt Institute (2003). Individual Asphalt Binder Tests.

